# Screening for Depressive Symptoms among Patients Attending Specialist Medical Outpatient Clinics in a Tertiary Hospital in Southern Nigeria

**DOI:** 10.1155/2018/7603580

**Published:** 2018-10-18

**Authors:** Sandra N. Ofori, Frances N. Adiukwu

**Affiliations:** ^1^Department of Internal Medicine, University of Port Harcourt, East-West Road, Choba, Rivers State, Nigeria; ^2^Department of Neuropsychiatry, University of Port Harcourt, East-West Road, Choba, Rivers State, Nigeria

## Abstract

This study aimed to determine the prevalence of unrecognized depressive symptoms and its associated risk factors among patients with diabetes and/or hypertension attending medical outpatient clinics of a tertiary health centre in southern Nigeria. A cross-sectional study design was employed to assess 200 randomly selected patients attending the clinics. Questionnaires were administered to obtain sociodemographic and medical history data. The perceived stress scale (PSS) was used to determine the presence of subjective psychological stress and PHQ-9 was used to screen for depression. The prevalence of depressive symptoms was 54.9% with 16.5% categorised as having major depression. After adjusting for confounding variables, age younger than 60 years was associated with less odds of having depressive symptoms (AOR 0.32, 95% CI 0.17, 0.62; p=0.001), while only significant psychological stress increased the odds of having depressive symptoms (AOR 2.78, 95% CI 1.37, 5.64; p=0.005). The prevalence of depression among the study participants is high and has the potential to significantly impact the control of their disease and ultimately contribute to the high cardiovascular risk faced by this population.

## 1. Introduction

Affective disorders (most commonly depressive and anxiety disorders) are among the most prevalent disorders seen in medical practice [1]. Depression, currently the 4th leading cause of global disease burden (GBD), is projected to be the 2nd leading cause of GBD after cardiovascular disease by 2020 [2, 3]. In 2010, major depressive disorder ranked as the second specific leading cause of global years lived with disability (YLD). This ranking has remained relatively unchanged since 1990 [4]. The global point prevalence of major depressive disorder (MDD) was estimated to be 4.7% by Ferrari et al. in a meta-analysis [5]. The World Health Organization estimated that 350 million people globally are affected by depression and this figure is on the rise [6].

Cardiovascular disease (CVD) is currently the leading cause of morbidity and mortality globally, with about 80% of all CVD mortality occurring in low- and middle-income countries including Nigeria [7]. This is partly due to the increasing prevalence in this region as a consequence of the epidemiologic transition, and the lack of adequate healthcare resources to tackle this highly complex disease condition. Cardiovascular disease can manifest acutely in which the first presentation is a sudden event or even sudden death. However, more commonly, with advances in diagnosis and treatment, affected individuals end up living with its consequences as a chronic medical condition. Major risk factors for CVD include hypertension and diabetes mellitus which themselves are chronic medical conditions that often require lifelong treatment.

The relationship between CVD and depression is bidirectional [8]. Depression is a major risk factor for CVD in the same way that diabetes and hypertension increase the risk for CVD. Furthermore, the prognosis is worse when people have comorbid depression and CVD. It has been demonstrated that, among patients with type II diabetes mellitus, the prevalence of major depressive episode (MDE) is higher with subsequent worsening of quality of life of the patients [9]. Similarly, 30% of patients with ischaemic heart disease and 38% of patients with arterial hypertension have been shown to have clinically significant depressive symptoms with increased risk of negative prognostic events (including all-cause mortality) [10].

Depressive symptoms and depression are still underrecognised and underdiagnosed in primary healthcare settings as well as specialist internal medicine clinics. This ultimately leads to prolonged suffering on the part of the patient and repeated utilization of healthcare services [11]. Family physicians rate depressed patients as more difficult to diagnose and treat. The presence of vague somatic complaints increases hospital visits but they also have much higher rates of defaulting from treatment follow-up [12–14]. The emotional impact of a CVD is often overlooked by clinicians and this requires attention especially now in this era of biological, psychological, and social approach to the management of all patients [15].

Hypertension is the leading risk factor for CVD in sub-Saharan Africa. Diabetes mellitus is a chronic and potentially debilitating medical condition that exerts a huge burden not only on the patient and healthcare system but also on the economy. In addition, it is a leading risk factor for CVD. These two chronic medical illnesses account for a high prevalence of all medical conditions seen in the University Port Harcourt Teaching Hospital Nigeria [8]. This study was therefore designed to investigate the prevalence of depressive symptoms in patients living with diabetes mellitus and/or hypertension and attending the specialist medical outpatient clinics of the hospital and who have had no previous contact with mental health physicians. We further sought to describe the risk factors associated with the presence of depressive symptoms in these patients.

## 2. Methods

### 2.1. Study Design

This was a cross-sectional hospital-based study carried out over a one-year period (March 2017 to February 2018) at the medical outpatient clinics of the University of Port Harcourt Teaching Hospital, Rivers State, Nigeria. The hospital is a 900-bed facility and serves as the main tertiary referral centre for Rivers State and the neighbouring south-south states of the Niger Delta. Rivers State is one of the 36 states and is in the south-south geopolitical zone of Nigeria and currently occupies an area of 1,077 square kilometers with a population of 5,198,716 people [16].

### 2.2. Sampling

A simple random sampling method was used and study participants were selected from the outpatient register with a referral diagnosis of diabetes mellitus and hypertension. Patients who present to the clinic are referred from the general outpatient clinic or following hospital admission for an acute presentation. The diagnosis of diabetes mellitus and/or hypertension was made following standard criteria by the referring physicians. The 2 clinics (endocrine and cardiovascular) register about 8 newly referred patients each clinic day, which is once a week. These patients had a study number allocated to them. The first was randomly selected and then every 3rd person was invited to participate and if they met the study criteria and gave informed consent, they were recruited. An informed consent form was given to all potential participants detailing the study's aim, procedure, and outcome. Patients were included if they were 18 years and older, were diagnosed with either hypertension, diabetes mellitus, or both, had not suffered complications related to either condition like stroke, congestive heart failure, kidney failure, or limb amputation, and consented to participate. Patients who had substance abuse problems, known neuropsychiatric disorders, or previous treatment by a clinical psychologist or psychiatrist were excluded. A total of 200 adults were included in the study.

### 2.3. Ethical Considerations

This study was approved by the ethical board of the University of Port Harcourt Teaching Hospital and carried out in keeping with the Helsinki Principles for human research. Participants either signed or thumb-printed a consent form. The purpose of the study was explained and the participants were reassured that the data collected was confidential and anonymised. They were also made to understand that participation was entirely voluntary and if they chose to decline or withdraw from participation at any time, there would be no negative consequences.

### 2.4. Study Procedure

Two trained research assistants administered the study instruments to each participant. The questionnaires were administered in English. The participants spoke English or had a relative with them who could. Participants were asked using a structured questionnaire about life style risk factors based on known risk factors for cardiovascular disease. History of alcohol use was assessed by asking each respondent if on any typical day they could consume at least 1 drink for females and 2 drinks for males, for which they answered “yes” or “no.” A family history of hypertension, diabetes, and cerebrovascular disease was also obtained. Each participant's weight was measured with a Seca mechanical weighing scale to the nearest 0.5 kg with the subject wearing only light clothing, and height was measured with the vertical ruled bar attached to the scale. The participant was asked to stand with their feet together without shoes or head gear, back and heel against the vertical bar, and the height reading was taken to the nearest 0.5 cm. Body mass index was calculated as body weight in kilograms divided by the square of the height in meters and classified according to the WHO criteria as normal weight (18.5-24.9 kg/m2), overweight (25–29.9 kg/m2), or obese (more than 30.0 kg/m2). Blood pressure was measured using a mercury sphygmomanometer (Accosson) with an appropriate cuff size on the patients' right arm in the seated position with feet on the floor after a five-minute rest. The first and fifth Korotkoff sounds were used to obtain the systolic and diastolic blood pressures, respectively. The average of two blood pressure measurements taken 5 minutes apart was used.

The presence of subjective psychological stress was enquired from each respondent using item 3 of the perceived stress scale (PSS) [17] and scored on a Likert scale of 0,1,2,3, and 4. Scores of 3 and 4 were considered significant psychological stress. The 9-item Patient Health Questionnaire (PHQ-9) was used to assess the presence and severity of depressive symptoms and major depression. PHQ-9 is based on the DSM-IV criteria for major depressive disorder and has been shown to be both a reliable and valid assessment tool [18]. Patients rated the frequency of experiencing the listed depressive symptoms on a scale of 0-3. A total score of 0 to 27 correlating with the severity of depression was calculated. Depression severity was grouped into none (score 0-4), minimal symptoms (score 5-9), and 10 or more as major depression. This was subcategorised as mild depression (score 10-14), moderately severe depression (score 15-19), and severe depression (score of greater than 20).

### 2.5. Statistical Analysis

This was performed with Statistical Package for the Social Sciences version 21.0 (SPSS Inc., Chicago, IL, USA). Continuous variables were expressed as mean (standard deviation) and categorical data as proportions and percentages. Logistic regression analysis was used to determine the factors associated with the outcome variable of interest which was presence or absence of depressive symptoms. P values less than 0.05 were considered statistically significant.

## 3. Results

### 3.1. Sociodemographic Characteristics and Medical History

A total of 200 patients were included in this study. Females constituted 52% of the study population, mean (SD) age was 57.9 (12.6), and 45.5% of them were above 60 years of age. Over half of the study population had a positive family history of diabetes or hypertension and 43% had a history of CVD in a first-degree relative. Only 10% were current smokers or had quit within last 1 year, and 35% consumed alcohol regularly. Majority did not engage in regular physical activity and 28% had a diet poor in fiber. The prevalence of diabetes mellitus was 60.5%: 45.5% had hypertension and 15% had both conditions coexisting. Most of the participants (75%) had significant subjective psychological stress (Table 1).

### 3.2. Clinical Characteristics of the Study Population

Up to 61% of the participants had BMI in the overweight and obese category. The mean (SD) PHQ9 score was 6 (4). The prevalence of depressive symptoms in the study participants was 59.5%, and 16.5% of study participants had major depression (11% had mild depression, 5% had moderately severe depression, and only one participant had severe depression) (Table 2).

### 3.3. Association of Study Variables with Depressive Symptoms

Being younger than 60 years reduced the odds of having depressive symptoms while having a positive family history of hypertension, family history of CVD in a first-degree relative, and significant psychological stress were associated with increased odds of depressive symptoms (Table 3).

In logistic regression, age less than 60 years reduced the odds while only significant psychological stress was associated with increased odds of depressive symptoms after adjusting for other variables (Table 4).

## 4. Discussion

The key findings from this study are that, in a select population of adults with uncomplicated hypertension and diabetes, the prevalence of depressive symptoms is high and both older age and subjective psychological stress were independently associated with depression.

Stress is a complex phenomenon that is built from the combination of exposure to a stressful event, the individual's perception of psychological stress resulting from that event, and the resultant biobehavioural responses to that stressful event. The PSS is widely used to assess how individuals perceive their current condition and higher scores have been shown to be associated with depressive symptoms related to life events [19]. Alkhathami et al., in their study of 368 primary healthcare patients with hypertension and diabetes, demonstrated that patients' feeling that their hypertension and diabetes were uncontrolled increased their levels of perceived stress and this was associated with depression and anxiety. This feeling was not associated with objective measures of control like glycated hemoglobin and measured blood pressure. The presence of subjective psychological stress was found to be a predictor of depression in subjects with chronic medical illness. The nature of this psychological stress was not part of the scope of this present study; however one cannot downplay the role having a first-degree relative with hypertension and cerebrovascular disease has to play on perceived stress, taking into account the role the immediate family plays in the care of their relative in the African setting and the subsequent care giver burden.

In our study 59.5% of the participants had significant depressive symptoms even though the majority (54%) were classified as having minimal symptoms and mild major depression. This was similar to the findings from a study conducted among patients presenting to the general medical outpatient clinics in a tertiary hospital in southwestern Nigeria. They found that, of their 272 participants, 47.8% had significant depressive symptoms with 49.2% being classified as mild and older age and having a clinical diagnosis of respiratory or cardiovascular disease was associated with depression. Further, they found that generally depression was underrecognized in their study population as none of the participants had depression as part of their diagnosis [11]. In northern Nigeria depression was found in 49.8% of patients attending the general outpatient clinic of a busy tertiary hospital. Significant associations were found between depression and age over 40 years, female gender, being married, poor education, and having a chronic medical condition including hypertension and diabetes mellitus [20]. Another study by Igwe et al. in southeastern Nigeria found high rates of depression among patients with diabetes and hypertension using the Mini International Neuropsychiatric Interview and they showed a significant association between depression in CMI and the presence of suicidality [21].

The association between depression and chronic medical illnesses, though not fully understood, might be mediated through behavioural mechanisms in which the limitations brought about by the medical illness on one's way of life and activities lead to decrease in pursuit of rewarding activities and subsequently the presence of anhedonia and other depressive symptoms. This disability brought about by chronic medical illness has been shown to be the most important determining factor of late life depression [22]. Other high risk behaviours for CVD such as problem alcohol drinking and physical inactivity have also been associated with the presence of depressive symptoms [23].

Major depression has been shown to worsen the outcome of CMI in terms of hospital readmission and mortality [24]. Depression increases the overall burden of disease in patients with chronic medical illness, as well as a decrease in the overall quality of life. The presence of depression increases days missed from work, restricts activity, and increases health service use and cost [12, 25]. We cannot be ignorant of the fact that, in Nigeria, being screened for a “psychiatric” condition or referred to the psychiatrist can generate conflict within the patient [26] and indeed within the physician [27] as well, thus contributing to the physician inertia to accurately recognise and address comorbid depression in medical patients. One way to mitigate this problem is by employing the use of liaison psychiatry. Patients with chronic medical illnesses without psychiatric disease but who have been identified to have subjective psychological stress by scoring high on the PSS can get reviewed by the liaison psychiatrist right there in the medical outpatient clinic. This integrated model if used in a tertiary institution setting will help overcome the stigma of a referral to psychiatric clinics, improve their pathway to care, and thereby improve the holistic management of the patient [28].

## 5. Conclusion

This study shows a high prevalence of depressive symptoms among patients presenting to medical outpatient clinics. Hypertension and diabetes are two very important risk factors for CVD and their control is mandatory for favorable cardiovascular outcomes. Depression as a comorbid condition can impact negatively achieving control and thus influence the manifestation of CVD. On the other hand, the stress of living with chronic medical conditions such as these may increase the risk of depression as well. The need to screen all patients living with chronic medical illnesses for depression and adoption of integrated care models for the management of these patients cannot be overemphasized.

### 5.1. Study Limitations

This study included patients with recently diagnosed diabetes mellitus and/or hypertension. The duration of the illness could therefore not be accurately ascertained as both conditions may go undiagnosed for many years. The cross-sectional nature of the study only allows the assessment of associations and cannot allow us to determine whether the depressive symptoms preceded the diagnosis or vice versa. Thirdly, the sample size, although based on the number of new patients seen at the clinic, is relatively small. Larger multicentre studies are required in order to allow generalisation of the findings to the larger population of Nigerian patients living with diabetes mellitus and/or hypertension.

## Figures and Tables

**Table 1:** Sociodemographic/medical history of study participants.

**Variable **	**Frequency (n=200)**	**Percentage**
Female	104	52.0
**Age Mean ± SD (years)**	57.96±12.63	
≤ 60 years	109	54.5
>60 years	91	45.5
**Family history of diabetes in first degree relative**	120	60.0
**Positive history of hypertension in first degree relative**	116	58.0
**Family history of CVD in first degree relative**	86	43.0
**Family history of Asthma**	1	0.5
**Family history of cancer**	1	0.5
**Smoking status**		
Never smoked	180	90.0
Current smoker	20	10.0
**Alcohol consumption**		
Never consumed alcohol	130	65.0
Consumes alcohol regularly	70	35.0
**Engages in physical activity less than recommendation**	180	90.0
**Inadequate Dietary fiber consumption**	56	28.0
**Diagnosed with diabetes**	121	60.5
**Diagnosed with hypertension**	91	45.5
**Co-morbid hypertension and diabetes**	30	15.0
**Significant subjective psychological stress**	150	75.0

**Table 2:** Clinical characteristics of 200 study participants.

**Variable **	**Value **
**Weight** (Kg) Mean ± SD	71.91±15.7
**Height** (m) Mean ± SD	1.63±0.06
**BMI** (Kg/m^2^) Mean ± SD	27.15±5.38
**BMI category**	
Underweight n (%)	1 (0.5)
Normal weight n (%)	77 (38.5)
Overweight n (%)	62 (31.0)
Obese n (%)	60 (30.0)
**Systolic BP (mmHg)** Mean ± SD	130±21.60
**Diastolic BP (mmHg)** Mean ± SD	80±12.79
**PHQ9** Mean ± SD	6.02±4.14
**Depression status**	
Depressed n (%)	119 (59.5)
**Level of depression**	
**Normal** n (%)	81 (40.5)
PHQ9 5-9 Minimal symptoms n (%)	86 (43.0)
PHQ9 10-14 Mild n (%)	22 (11.0)
PHQ9 15-19 Moderately severe n (%)	10 (5.0)
PHQ9 ≥20 Severe depression n (%)	1 (0.5)

**Table 3:** Association of study variables with depression.

**Variable**	**Depression status**		**χ**2	**p-value**
	**Normal 81**	**Depressed 119**		
	**n (**%**)**	**n (**%**)**		
**Gender **				
(i) Male	36 (44.4)	60 (50.4)	0.690	0.406
(ii) Female	59 (55.6)	59 (49.6)	0.690	0.406
**Age**				
(i) ≤60 years	58 (71.6)	51 (42.9)	16.062	**<0.001** **∗**
(ii) >60 years	23 (28.4)	68 (57.1)		
**Positive history of hypertension in first degree relative**	36 (44.4)	80 (67.2)	10.269	**0.001** **∗**
**Family history of CVD in first degree relative**	25 (30.8)	61 (57.3)	8.180	**0.004** **∗**
**Family history of diabetes in first degree relative**	45 (55.6)	75 (63.0)	1.120	0.290
**Family history of Asthma**	0 (0.0)	1 (0.8)	0.684	0.408
**Family history of cancer**	0 (0.0)	1 (0.8)	0.684	0.408
**Current smoker**	8 (9.9)	12 (10.1)	0.002	0.962
**Consumes Alcohol**	31 (38.3)	39 (32.8)	0.640	0.424
**Engages in physical activity less than recommendation**	73 (90.1)	107 (89.9)	0.002	0.962
**Inadequate Dietary fiber consumption**	25 (30.9)	31 (26.1)	0.554	0.457
**Diagnosed with diabetes**	50 (61.7)	71 (59.7)	0.086	0.769
(i) FBG mean (SD)	9.36±3.88	9.69±5.28	0.375^#^	0.708
(ii) FBG <7 mmol/l	16 (35.6)	29 (64.6)	0.703	0.420
(iii) FBG ≥7 mmol/l	35 (46.1)	41 (53.9)		
**Diagnosed with hypertension**	35 (43.2)	56 (47.1)	0.288	0.592
(i) SBP mean (SD)	129.8(20.82)	134.2(22.04)	1.389^#^	0.166
(ii) DBP mean (SD)	81.09 (14.73)	82.04 (11.35)	0.517^#^	0.605
**Significant subjective psychological stress**	51 (63.0)	99 (83.2)	10.520	**0.001** **∗**
**BMI category**				
(i) Underweight/Normal	27 (33.3)	51 (42.9)	1.838	0.175
(ii) Overweight/Obese	54 (66.7)	68 (57.1)		

**Table 4:** Predictors of depression using logic regression analysis.

**Variable **	**OR**	**95**%** CI**	**P value**	**Adjusted OR**	**95**%** CI for AOR**	**P value**
**Age group ≤60 years**	0.29	0.16, 0.54	**<0.001**	0.32	0.17, 0.62	**0.001**
**Positive history of hypertension in first degree relative**	2.56	1.43, 4.59	**0.002**	1.70	0.86, 3.38	0.130
**Positive history of CVD in first degree relative**	2.36	1.30, 4.26	**0.005**	1.33	0.66, 2.71	0.424
**Significant subjective psychological stress**	2.91	1.51, 5.62	**0.001**	2.78	1.37, 5.64	**0.005**

## Data Availability

The SPSS data used to support the findings of this study are available from the corresponding author upon request.
